# The effect of different atmosphere absolute hyperbaric oxygen on the expression of extracellular histones after traumatic brain injury in rats

**DOI:** 10.1007/s12192-020-01137-6

**Published:** 2020-07-23

**Authors:** Fang Liang, Lei Sun, Jing Yang, Xue-Hua Liu, Jing Zhang, Wan-Qiu Zhu, Lu Yang, Ding Nan

**Affiliations:** Department of Hyperbaric Oxygen, Beijing Chao-Yang Hospital, Capital Medical University, 8 South Gongti Road, Chao-Yang District, Beijing, 100020 China

**Keywords:** Hyperbaric oxygen, Traumatic brain injury, Extracellular histones

## Abstract

By observing the dynamic changes of extracellular histones H1, H2A, H4, and NF-κB expression in brain tissues after brain injury in rats, we explore the association among the expression of extracellular histones H1, H2A, H4, and NF-κB following traumatic brain injury (TBI), as well as the effect of different atmospheres absolute hyperbaric oxygen (HBO) intervention on the expression and possible mechanisms. A total of 120 SD rats were randomly divided into 4 groups: Sham-operated (SH), TBI (traumatic brain injury) group, traumatic brain injury and hyperbaric oxygen treatment 1.6ATA (TBI + HBO1) group, and traumatic brain injury and hyperbaric oxygen treatment2.2ATA (TBI + HBO2) group, with 30 rats in each group. The rats in each group were then randomly divided into five smaller time-specific sub-groups: 3 h, 6 h, 12 h, 24 h, and 48 h after surgery. TBI models were established, and the brain tissue around the lesion was taken at different time points. On the one hand,we detected the level of local histones H1, H2A, H4, and NF-κB by RT-PCR and Western Blot. On the other hand, we used immunohistochemical methods to detect the expression of NF-κB, while using the TUNEL method to observe the cell apoptosis in experimental groups after brain injury. Extracellular histones H1, H2A, H4, and NF-κB proteins were highly expressed at 3 h, then with a slight fluctuation, reached to peak at 48 h after the injury. HBO can affect the expression of histones H1, H2A, H4, and NF-κB. The decline of each indicator in the 1.6ATA group was significantly lower than that in the 2.2ATA group, especially within 6 h (*P* < 0. 05). In addition, NF-κB expression was consistent with the pathological changes of apoptosis in experimental groups. Hyperbaric oxygen therapy with relatively low pressure (1.6ATA) at the early stage can significantly inhibit the expression of extracellular histones H1, H2A, H4, and NF-κB around the lesion, reduce the apoptosis of nerve cells, and thus play an important role in alleviating secondary brain injury.

## Introduction

In recent years, the incidence of traumatic brain injury (TBI) caused by motor vehicle accidents, falls, violence, and blunt trauma has increased.(Ng & Lee, 2019) WHO stated that TBI will be the most frequent disorder in 2020. Damage to brain function due to trauma may be permanent, leading to emotional, cognitive, and motor disturbances. The primary injury is not reversible. Most of the novel and effective therapeutic strategies focus on the process of the secondary injury initiated by immune activation. A series of cellular and molecular changes play an important role in these cascades, resulting in excitotoxicity neuroinflammation and ultimately apoptotic cell death of neurons and glia.(Jassam et al., 2017).

In recent years, extracellular histones have been identified as a crucial factor in the subsequent inflammatory process of tissue injury caused by hypoxia and ischemia. Histones are the basic structural proteins of chromosomes found in eukaryotic cell nuclei, which consist of two functional subgroups, “core” (H2A, H2B, H3, and H4) and “linker” (H1 and H5) proteins. Core proteins are wound around DNA form nucleosomes and linker histones join adjacent nucleosomes together.(Xu et al., 2015; Andrews & Luger, 2011) The release of histones into the extracellular space might come from apoptotic or necrotic cells following injury, or as a component extruded from neutrophils into the extracellular space to form the fibrous networks named neutrophil extracellular traps (NETs).(Rosin & Okusa, 2012; Silk et al., 2017) Extracellular histones possess direct cytotoxicity, induced endotheliocyte damage, and platelet aggregation. More importantly, they could act as new members of damage-associated molecular pattern molecules (DAMPs), which activate systemic immune responses after traumatic injury and leading to cell apoptosis.(Semeraro et al., 2011; Takeda & Akira, 2015) Most of the research in this field has focused on liver and lung injury; however, there is a limited study within TBI.

Hyperbaric oxygen therapy (HBOT), in which the patient inhales pure oxygen or a high concentration of oxygen in an environment maintained above one atmosphere, is widely used clinically for TBI. A great deal of research shows that HBOT can reduce secondary brain damage in brain injury by improving oxidative metabolism and reducing neuro-inflammatory responses. Eilam Palzur et al. found that the neuroprotective effect of HBOT may represent the consequence of preserved mitochondrial integrity and subsequent inhibition of the mPTP and reduction of the mitochondrial pathway of apoptosis.(Palzur et al., 2008). However, some reports put forward that replenishment of O_2_ might increase ROS production, exacerbating neuronal damage. HBOT in TBI was still lacking in-depth research on molecular mechanisms and a further optimized recommended scheme in which HBOT is administered.

In the present study, we aimed to understand how different atmosphere absolute (ATA) hyperbaric oxygen (HBO) intervention influences the individual histones and to describe the molecular landscape of histone-mediated auto-immune responses and apoptosis in peri-lesion cells, providing new clues about the therapeutic mechanisms underlying the extensive and standard applications of HBO.

## Materials and methods

### Experimental animals

Experimental Sprague Dawley rats (8-weeks old, 250 to 500 g, male and female) were provided by the Experimental Animal Center of Capital Medical University. Animals were housed in a controlled environment at 20 °C with food and water ad libitum. Rats were fasted for 8 h and deprived of water for 2 h prior to surgery. All experiments were performed in accordance with the National Institutes of Health Guide for the Care and Use of Laboratory Animals.

### Instruments

The main instruments used included an HPD-1700 Fluid Percussion Device (DRAGONFLY R&D INC), an ASI Small Animal Stereotaxic Instrument (ASI), and a DWC150-300 Pure Oxygen Animal Experiment Chamber (Yangyuan Medical Oxygen Cabin Factory of Shanghai 701 Institute).

### Grouping of experimental animals

#### Experimental groups

A total of 120 SD rats were randomly divided into 4 groups: Sham-operated (SH), TBI (traumatic brain injury) group, traumatic brain injury and hyperbaric oxygen treatment 1.6ATA (TBI + HBO1) group, traumatic brain injury and hyperbaric oxygen treatment 2.2ATA (TBI + HBO2) group, with 30 rats in each group. The rats in each group were then randomly divided into five smaller time-specific sub-groups: 3 h, 6 h, 12 h, 24 h, and 48 h after surgery.

#### Rat traumatic brain injury model

Rats were anesthetized by intraperitoneal injection of 10% chloral hydrate (350 mg/kg) and fixed onto the stereotaxic instrument in a prone position. The disappearance of corneal reflex and righting reflex were used as indices of successful anesthesia. The rat’s head was disinfected and the skin of the right cranial top cut under sterile conditions to expose the skull. A 3-mm diameter hole was drilled through the right parietal bone (4 mm from the skull herringbone and 3 mm lateral from the sagittal suture) while leaving the dura mater intact. A 3-mm diameter percussion tube adhered to the drill hole with cyanoacrylate adhesive glue and zinc phosphate cement, and then the surrounding skin was sutured. The reservoir tube of the hydraulic percussion instrument was filled with 37 °C physiological saline, and the angle and weight of the percussion hammer were preset. The reservoir tube was connected to the percussion tube, and the hammer was released to let the saline solution impact the cortical surface. Signs of successful percussion included edema and hemorrhage as evidenced by purple-red discoloration and bulging of the dura mater. After percussion injury, the percussion tube was removed, the skull hole closed with bone wax, and the incision completely sutured.

#### Sham surgery

Anesthesia, disinfection, skin incision, and skull drilling were performed as described for the TBI model except that the skull hole was closed with bone wax without percussion injury prior to suturing. The rats in the SH group underwent laminectomy without SCI or HBO treatment.

### HBO therapy

The chamber was perfused with pure oxygen for 5 min, and then the pressure was increased to 1.6 and 2.2 atm over 10 min respectively. The animals inhaled hyperbaric oxygen for 60 min at stable pressure and then decompressed for 15 min. During HBO treatment, the chamber was continuously ventilated at 8–10 L/min to prevent CO2 accumulation and maintain the oxygen concentration at 95%. The chamber temperature was maintained at 22 to 26 °C, and the humidity between 40 and 50%. Animals in the TBI groups were placed in the chamber for the same duration but inhaled oxygen at atmospheric pressure. In both groups, HBO therapy was administered daily at 11 am for consecutive 14 days.

### Evaluation of neural function and sample collection

The recovery of neural function was evaluated by modified neurological severity (mNSS) scores at 3 h, 6 h, 12 h, 24 h, and 48 h after surgery.(Chen et al., 2001). The mNSS score test includes the assessment of motor, sensory, balance, and reflex functions. The tests were conducted by observers who were blinded to the experimental conditions and treatments. The mNSS score was graded on a scale of 0 to 14, normal score 0; maximal deficit score 14. One score point is awarded for the inability to perform the test or for the lack of a tested reflex: 10–14, severe; 5–9, moderate; and 1–4, mild injury.

Following the evaluation of neural function, the animals were sacrificed using chloral hydrate. The sternum cut to expose the heart, and the left ventricle injected with 0.2 ml 1% heparin sodium. Aortic vessel was performed via the left ventricle and the auricula dextra was cut. The blood vessels were quickly perfused with 150 ml normal saline. After perfusion, the rats were decapitated to obtain brain tissue from cranium without traumatization. The brain tissue of the area peripheral to the injury was carefully extracted, divided into two parts along the midline of the injury. One part was stored in 4% paraformaldehyde for HE stain, immunohistochemical, and TUNEL method, the other stored in a − 80 °C refrigerator for polymerase chain reaction (PCR) and Western blot analysis.

### Laboratory tests

#### Reverse transcription-quantitative polymerase chain reaction

Total RNA was extracted from the frozen brain tissue by routine methods and quality tested by 1% agarose gel electrophoresis. Reverse transcription was performed using the TIANScript RT Kit. A 12-to-18-μl reaction mixture containing 3 μl of template RNA and 0.5 μl of primer (50 μM) was prepared, heated to 70 °C for 5 min for denaturation, and then quickly cooled on ice for over 2 min. The template RNA/primer denatured solution was precipitated by several seconds of centrifugation. The reverse transcription (RT) reaction system included template RNA/primer pellet, 5 μl of 5 × M-MLV buffer, 1.25 μl of dNTP mixture (10 mm of each nucleotide), 25 U of RNase inhibitor (40 U/μl), 200 U of M-MLV (200 U/μl), and RNase-free dH2O in a total volume of 25 μl. The RT reaction was run at 42 °C for 2 h and 95 °C for 15 min. Real-time PCR was performed using a SYBR FAST qPCR Kit (KAPA Biosystems, USA). The amplified Histone H1, Histone H2A, Histone H4, TLR4, and NF-κB were 182, 125, 94, 105, and 113 bp, respectively (Table 1). The 2-ΔΔCT method was used to calculate relative expression levels of target mRNAs. GAPDH was used as internal control.

**Table 1:** Primers for qRT-PCR

Primer sequences		Probes	Sizes of PCR products
Histone H1	Forward primer	5′-CGTAAGAGATGCCGCGAAGA-3′	182 bp
	Reverse primer	5′-ATGGTCGAGCGCTTGTTGTA-3′	
HistoneH2A	Forward primer	5′-CCAGGATCTCCCGACTCCTT-3′	125 bp
	Reverse Primer	5′-GAAGTGGCGGGGTTGAAACT-3′	
HistoneH4	Forward primer	5′-CGTACCCTCTATGGCTTCGG-3′	94 bp
	Reverse primer	5′-AAAAGTTGGGTGGAAGCAAAGC-3′	
TLR4	Forward primer	5′-GCCCTGTTGGATGGAAAAGC-3′	105 bp
	Reverse primer	5′-ATGGGTTTTAGGCGCAGAGT-3′	
NF-κB	Forward primer	5′-AGTCCCGCCCCTTCTAAAAC-3′	113 bp
	Reverse primer	5′-ATCACTTCAATGGCCTCTGTGT-3′	
Actin	Forward primer	5′-TATCCTGGCCTCACTGTCCA-3′	130 bp
	Reverse primer	5′-AAGGGTGTAAAACGCAGCTCA-3′	

#### Western blotting

Total protein was extracted from 20 μg of frozen tissue in 400 μl of protein lysis buffer by homogenization on ice. The homogenates were transferred to pre-chilled 1.5 ml EP tubes, placed on ice for 15 min for complete lysis, and centrifuged at 12000 rpm for 10 min at 4 °C. The supernatants were transferred to 0.5 ml centrifuge tubes and stored at − 20 °C. A 50-to-100-μg sample was mixed with 5 × loading buffer, heated in boiling water for 5 min, quickly cooled, and loaded onto a polyacrylamide stacking gel for electrophoresis. The stacking gel was run at 80 V and separation gel at 120 V. Separated proteins were then transferred to PVDF membranes. Membranes were washed with tris-buffered saline with 1% Triton (TBS-T) for 5 min and incubated with the primary antibody diluted in blocking solution at 4 °C overnight. Membranes were washed 3 times with TBS-T (5 min per wash) and then incubated for 2–3 h with horseradish peroxidase (HRP)-labeled secondary antibody (Jackson, USA) diluted in blocking buffer. Blotted membranes were washed again with TBS-T (three times, 5 min each) and bands visualized with ECL chemiluminescence reagent. Gray-scale images were analyzed using imaging software.

#### Immunohistochemistry

Tissue samples fixated with formalin, NF-KBp65 were investigated in immunohistochemical analysis. NF-KBp65 antibody (Rabbit 1:200) (Abcam, Lot No. Ab16502) were purchased from Abcam plc (Cambridge, UK).

Paraffin-embedded tissue samples were sectioned, placed in EDTA antigen retrieval solution, and heated by microwave. After cooling, the sections were washed three times (5 min/wash) with phosphate-buffered saline (PBS, pH 7.4) under shaking, incubated in 3% hydrogen peroxide solution for 25 min at room temperature in a dark environment to quench endogenous peroxidase activity, washed with PBS (three times for 5 min each), drip-coated with 3% BSA for 3 min, and then incubated with the pre-diluted primary antibody solution at 4 °C overnight. After washing in PBS, the sections were incubated with HRP-labeled secondary antibody at room temperature for 50 min. After washing and drying, DAB solution was added for staining. Color development time was controlled by microscopy observation. For qualitative evaluation, brown-yellow staining was considered positive. After color development was terminated by running water, the sections were counterstained with hematoxylin, dehydrated, and mounted.

#### TUNEL method

Tissue samples fixated with formalin were embedded in paraffin. Sections in the thickness of 4 mm obtained from the paraffin blocks were affixed to positively charged slides. Apoptotic cells in the sections were shown with the TUNEL (terminal deoxynucleotidyl transferase-mediated dUTP nick end labeling) method by using Apoptosis Kit (Roche, Basel, Switzerland). The sections were kept at 37 °C overnight. The sections that were deparaffinized by passing through the xylol and reduced alcohol sequences were washed in the PBS (Zhongke Wanbang Biotechnology, Beijing, China). The sections were then incubated in the incubator with the proteinase K (Zhongke Wanbang Biotechnology, Beijing, China), an enzyme for 15 min at 37 °C. 3% H2O2 prepared in the PBS was applied to the sections in a dark environment for 5 min to prevent the endogenous peroxidase activity. TdT enzyme was applied to the sections for 1.5 h, which were kept in the balancing buffer in the incubator for 10 min at 37 °C. In order to stop the reaction of the TdT enzyme, after applying the stopping/washing buffer in the kit for 20 min at room temperature, the sections were washed 3 times in the PBS. The anti-digoxigenin peroxidase enzyme was applied in the incubator at 37 °C. After washing in the PBS, 3.3′-diaminobenzidine (DAB) substrate was applied to the sections as chromogen. After the application for 30–45 min, the reaction in brown color was stopped with distilled water. After this stage, the sections were stained with methyl green, which was used as the opposite dye, and rapidly passed through butanol. The slides that were made transparent by applying xylol for 15 min in total were covered with neutral gum and prepared for review using a light microscope (NIKON CI-S).

#### Calculation of Immunohistochemical positive staining rates and apoptotic index

All evaluations were performed independently by two blind observers. The positive rates of immunohistochemical staining were evaluated randomly in 8–10 different fields for all individuals of different groups. The percent value of positively stained cells (as indicated by brown-yellow particles) was counted according to the staining intensity. The immunohistochemical positive staining rates were calculated using the formula 100% × (number of positive cell / number of the total cell).

The apoptotic index was determined independently by two blind observers by counting under × 400 magnification and randomly selecting 8–10 different fields for all individuals groups. The apoptotic index was calculated using the formula 100% × (TUNEL-number of positive cell nucleus / number of total cell nucleus).

### Statistical analysis

Statistical analysis was performed using SPSS23.0 software. All variables were first tested for normality. Measurement data are expressed as mean ± standard deviation. Group means were compared by ANOVA. *P* < 0.05 (two-tailed) was considered statistically significant for all tests.

## Results

### 1.6ATA HBOT decreased the mNSS score of the rats following TBI within 48 h

**Fig. 1:**
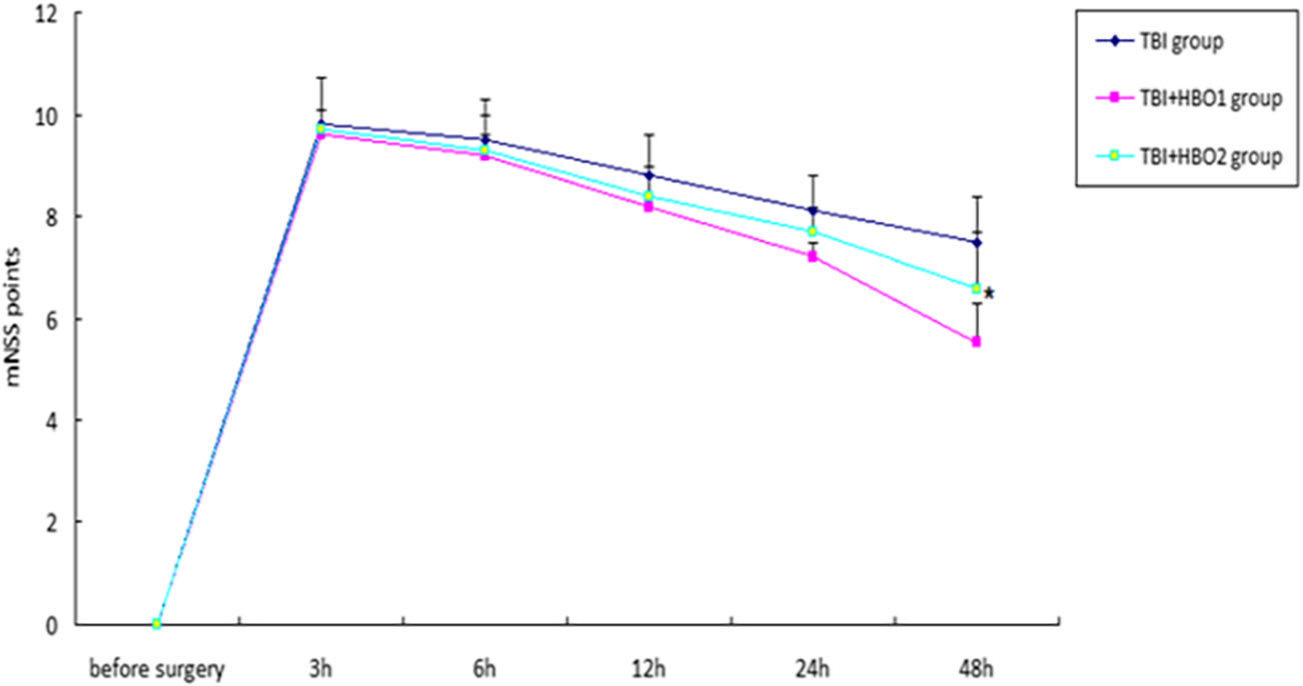
mNSS scores in the TBI groups and the TBI + HBO groups. Data are presented as the mean ± standard deviation.*P < 0.05, TBI + HBO1 groups vs. TBI groups

Neurological function changes in rats were evaluated using the mNSS score test. The mNSS scores in the SH groups were zero. The mNSS scores were significantly increased after surgery in both the TBI and TBI + HBO groups rats. The mNSS scores were significantly reduced in the TBI + HBO1 groups at 48 h compared with those in the TBI groups (*P* < 0.05), while there was no significant difference between the TBI and the TBI + HBO1 groups (Fig. 1). It suggested that 1.6ATA HBOT significantly improved neural functional recovery in the early stage of TBI.

### HBOT attenuated mRNA and protein expression of extracellular histone H1, H2A, and H4 following TBI. The decline of histones in the 1.6ATA groups was significantly lower than that in the 2.2ATA groups, especially in the early stages

We examined mRNA and protein expression of Histone H1, H2A, and H4 in different groups to explore the cytotoxicity of each individual histone and whether HBO protects neurons against the release of extracellular histones.

**Fig. 2:**
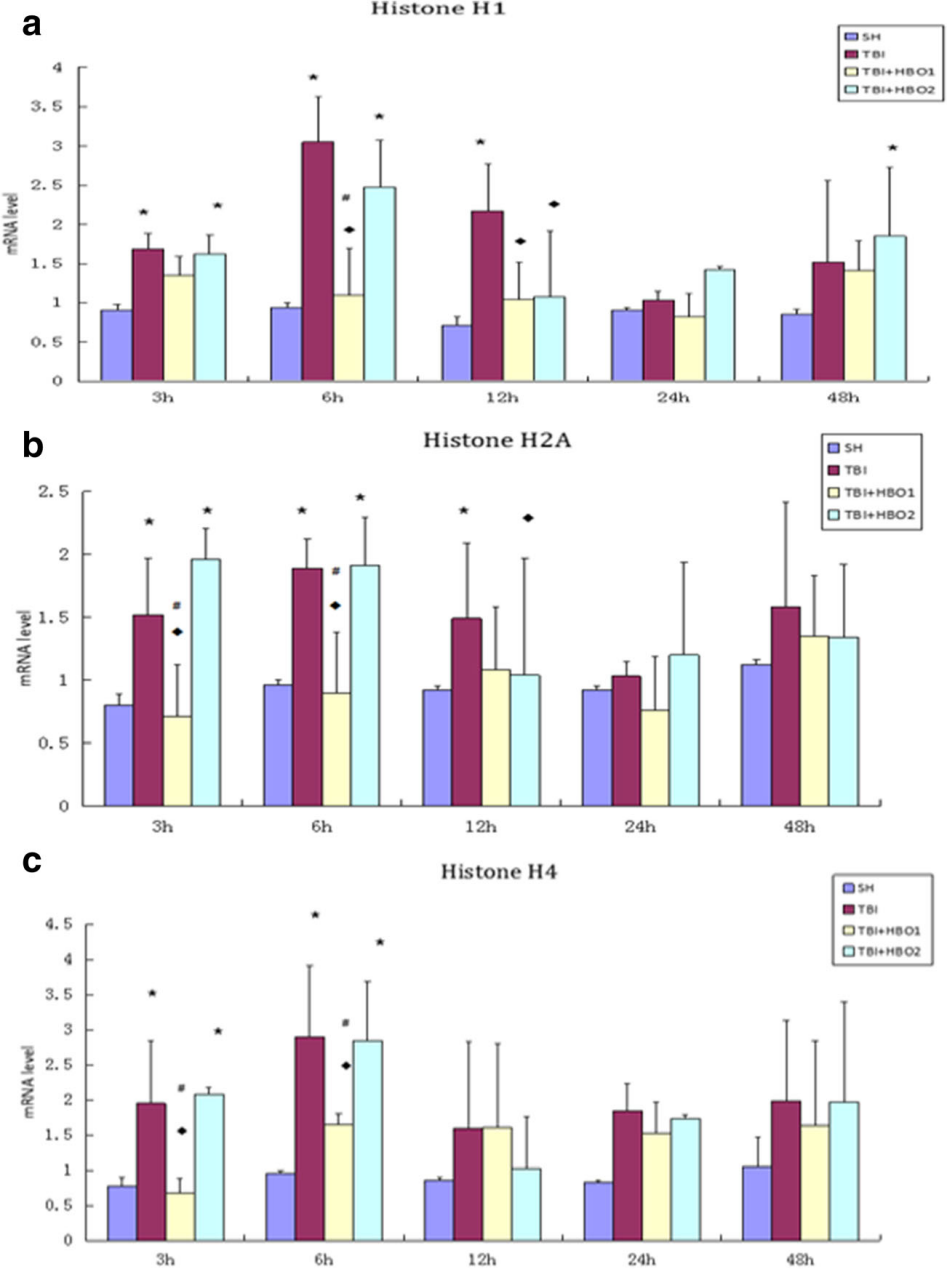
The effects of different ATA HBOT on the mRNA expression level of extracellular histones calculated by the 2-ΔΔCT method in experimental groups. (**a**) The quantification of histone H1 mRNA levels. (**b**) The quantification of histone H2A mRNA levels. (**c**) The quantification of histone H4 mRNA levels. *P < 0.05, TBI, TBI + HBO1 and TBI + HBO1 groups vs. SH groups; ♦ *P* < 0.05, TBI + HBO1 and TBI + HBO2 groups vs. TBI groups; ^#^*P* < 0.05, TBI + HBO1 groups vs. TBI + HBO 2 groups

Histone H1, as linker protein, is one of the principal chromatin components, which possess direct glial and neurotoxicity. As shown by our qRT-PCR results, the mRNA expression levels of extracellular Histone H1 apparently increased, peaking at 6 h after TBI, and decreasing by the intervention of 1.6ATA and 2.2ATA HBO significantly compared with the TBI groups at 6 h and 12 h respectively (*P* < 0.05) (Fig. 2A).

NET-derived H2A, the most prominent histone, induced direct damage to the endothelial cell. The mRNA expression levels of extracellular Histone H2A reached a peak at 3 h after TBI, and also decreased by the intervention of HBO significantly compared with the TBI groups (*P* < 0.05). 1.6ATA HBO intervention significantly reduced the mRNA level at 3 h and 6 h, while 2.2ATA HBO intervention declined at 12 h after TBI (*P* < 0.05) (Fig. 2B).

Histone H4 is the core protein of chromatin, abundant in intimal NETs. A previous study has demonstrated that NET-bound and free histone H4 exhibit strong cytotoxicity via interaction with the cytomembrane, contributing to vascular tissue damage.(Silvestre-Roig et al., 2019) Like H1, the mRNA expression levels of extracellular histone H4 reached a peak in the 6 h TBI group. 1.6ATA HBO intervention significantly reduced the mRNA level of histone H4 at 3 h and 6 h after TBI. There was no significant difference among the 2.2ATA HBO groups compared with TBI (*P* < 0.05) (Fig. 2C).

**Fig. 3:**
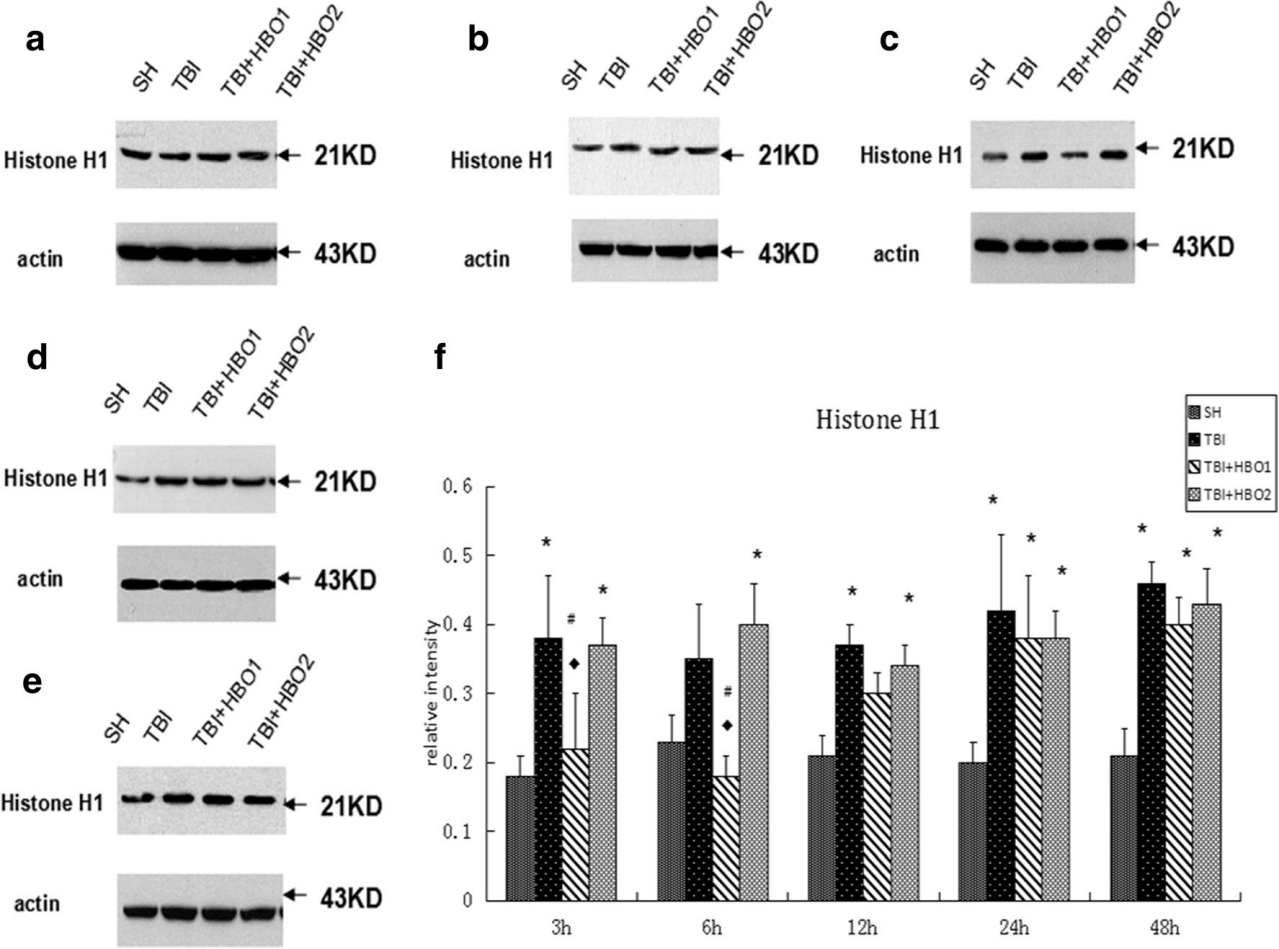
The effects of different ATA HBOT on the protein expression level of extracellular histone H1 detected by Western blots. (**a**) Representative Western blot bands of histone H1 in each group at 3 h after TBI. (**b**) 6 h, (**c**) 12 h, (**d**) 24 h, (**e**) 48 h (**f**) Relative densitometric quantification of histone H1.*P < 0.05, TBI, TBI + HBO1 and TBI + HBO1 groups vs. SH groups; ♦ *P* < 0.05, TBI + HBO1 and TBI + HBO2 groups vs. TBI groups; ^#^*P* < 0.05, TBI + HBO1 groups vs. TBI + HBO2 groups

**Fig. 4:**
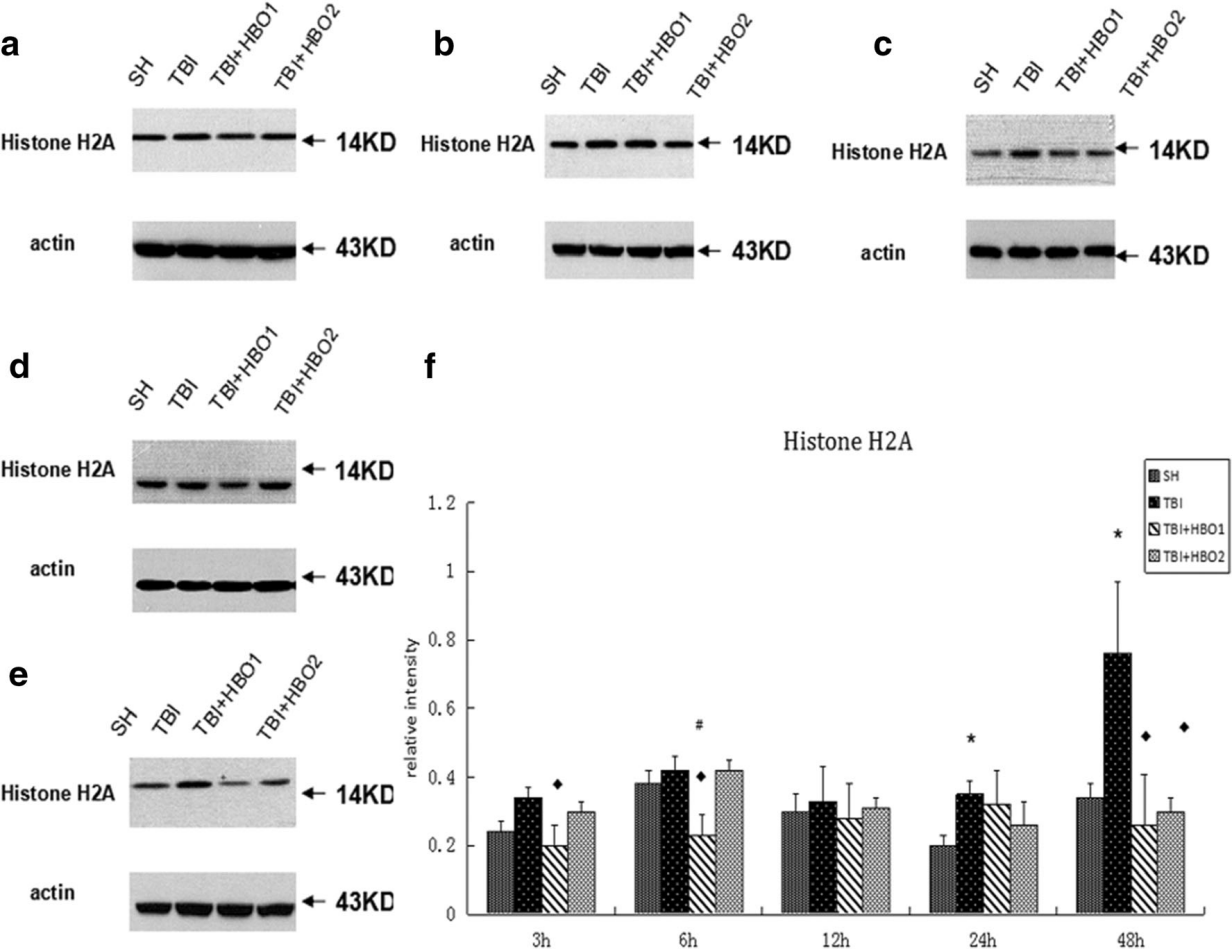
The effects of different ATA HBOT on the protein expression level of extracellular histone H2A detected by Western blots. (**a**) Representative western blot bands of histone H2A in each group at 3 h after TBI. (**B**) 6 h, (**c**) 12 h, (**d**) 24 h (**e**) 48 h (**f**) Relative densitometric quantification of histone H2A.*P < 0.05, TBI, TBI + HBO1, and TBI + HBO1 groups vs. SH groups; ♦ *P* < 0.05, TBI + HBO1, and TBI + HBO2 groups vs. TBI groups; ^#^*P* < 0.05, TBI + HBO1 groups vs. TBI + HBO2 groups

**Fig. 5:**
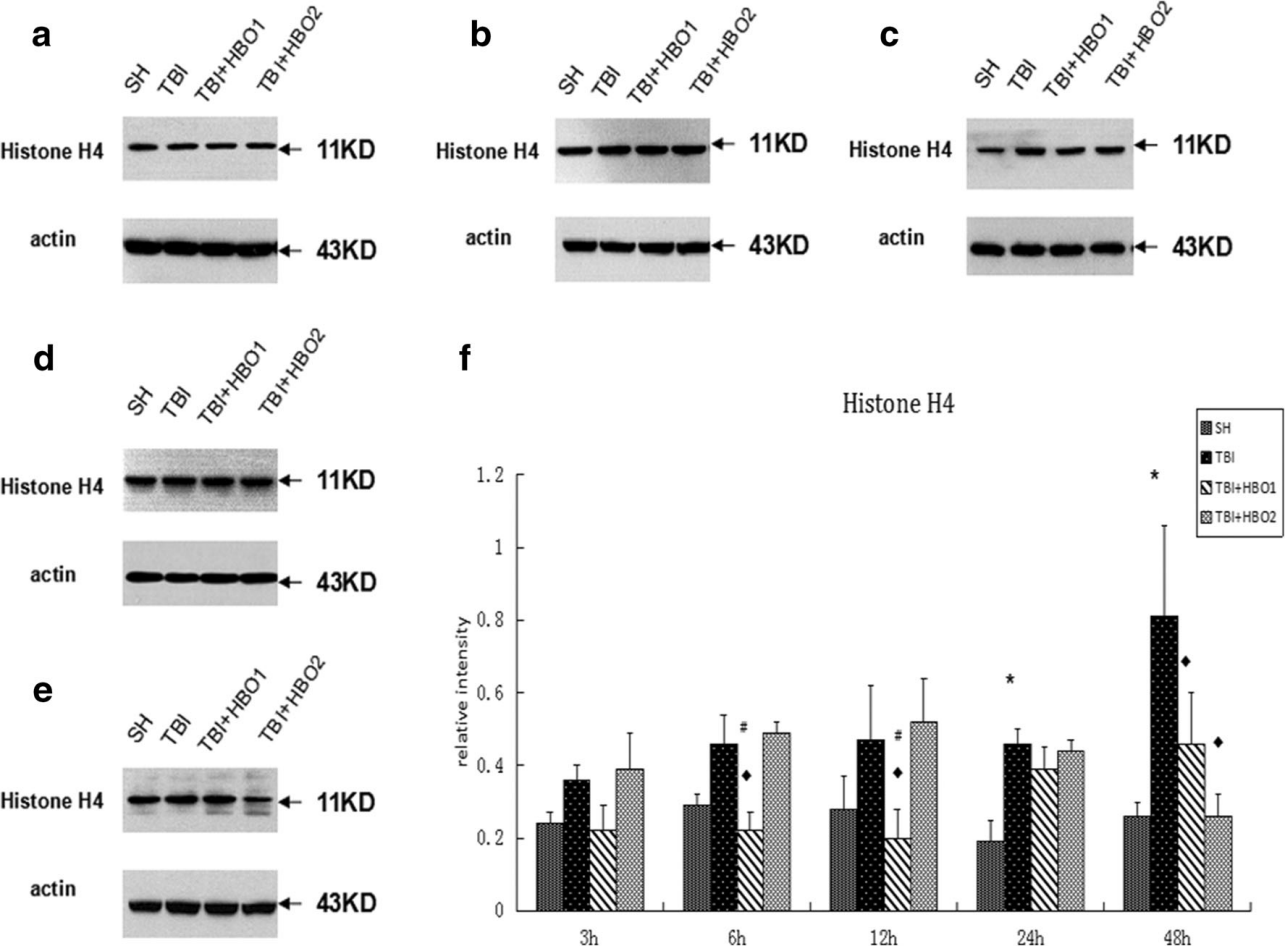
The effects of different ATA HBOT on the protein expression level of extracellular histone H4 detected by Western blots. (**a**) Representative western blot bands of histone H4 in each group at 3 h after TBI. (**b**) 6 h, (**c**) 12 h, (**d**) 24 h, (**e**) 48 h (**f**) Relative densitometric quantification of histone H4. *P < 0.05, TBI, TBI + HBO1, and TBI + HBO1 groups vs. SH groups; ♦ *P* < 0.05, TBI + HBO1, and TBI + HBO2 groups vs. TBI groups; ^#^*P* < 0.05, TBI + HBO1 groups vs. TBI + HBO 2 groups

Western blot analysis was performed to detect the protein levels of extracellular histones. In our study, it was shown that the level of histone H1 (Fig. 3), H2A (Fig. 4), and H4 (Fig. 5) increased gradually 3 h after TBI, then with a slight fluctuation, reached a significant peak in the 48 h TBI group (*P* < 0.05), indicating the release of histones from the nucleosome. 1.6ATA HBO intervention significantly reduced the level of histone H1, H2A, and H4 compared with TBI particularly at 6 h and 12 h (*P* < 0.05); however, 2.2ATA HBO intervention reduced histone H2A and H4 levels significantly at 48 h after TBI (*P* < 0.05).

Furthermore, we conducted a comparison of mRNA and protein levels between 1.6ATA HBO and 2.2ATA HBO groups to observe the optimal pressure of HBO to affect histones levels. Both mRNA and protein levels of histone H1, H2A, and H4 in 1.6ATA groups were lower than in the 2.2ATA groups, especially at 3 h and 6 h (*P* < 0.05) (Figs. 2, 3, 4, and 5).

Collectively, these data indicated that 1.6ATA HBO intervention could inhibit TBI-induced release of extracellular histones in brain tissue more efficiently compared with 2.2ATA in the early stages of injury.

### 1.6ATA HBO attenuated expression levels of downstream cytokine NF-κB p65 following TBI

With the exception of cytotoxicity, extracellular histones could activate the immune system in the secondary damage of injury. There is extensive evidence that histones may interact with TLR4 to initiate this immune response, which is responsible for the release of pro-inflammatory cytokines via TLR4/MyD88/NF-κB signaling pathways.(Bosmann et al., 2013; Wen et al., 2013). We wondered whether this signaling pathway was involved in the effects of HBOT, so the level of downstream cytokine NF-κB p65 in the experimental groups was measured.

**Fig. 6:**
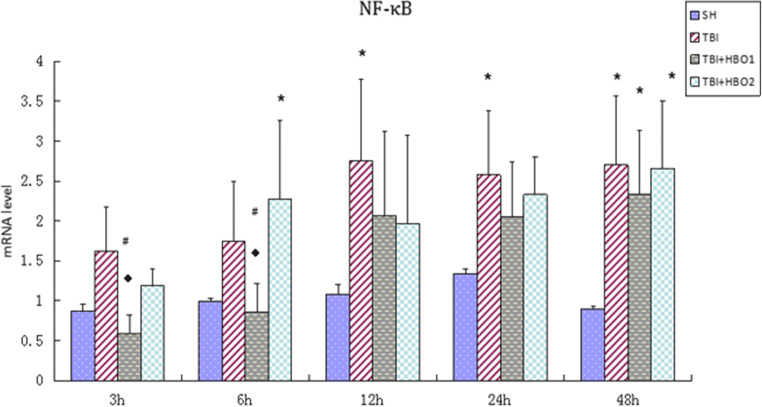
The effects of different ATA HBOT on the mRNA expression level of extracellular histones calculated by the 2-ΔΔCT method in experimental groups. *P < 0.05, TBI, TBI + HBO1, and TBI + HBO1 groups vs. SH groups; ♦ *P* < 0.05, TBI + HBO1, and TBI + HBO2 groups vs. TBI groups; ^#^*P* < 0.05, TBI + HBO1 groups vs. TBI + HBO 2 groups

**Fig. 7:**
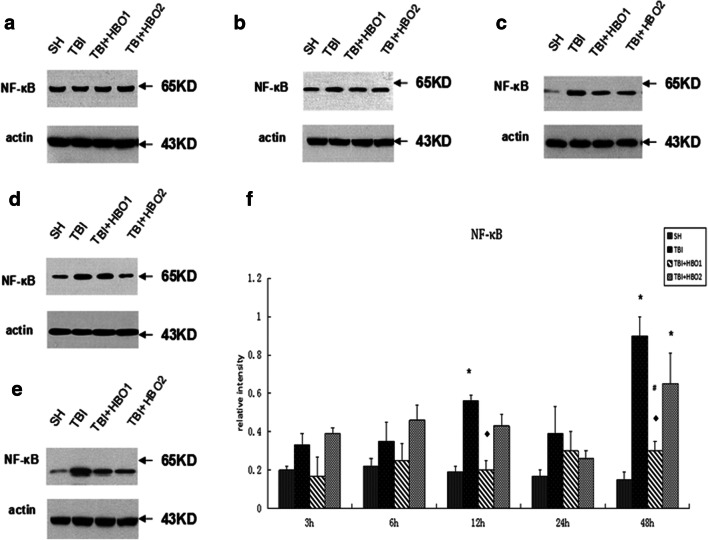
The effects of different ATA HBOT on the protein expression level of NF-κB detected by Western blots. (**a**) Representative western blot bands of NF-κB in each group at 3 h after TBI. (**b**) 6 h, (**c**) 12 h, (**d**) 24 h, (**e**) 48 h, (**f**) Relative densitometric quantification of NF-κB. *P < 0.05, TBI, TBI + HBO1, and TBI + HBO1 groups vs. SH groups; ♦ *P* < 0.05, TBI + HBO1, and TBI + HBO2 groups vs. TBI groups; ^#^*P* < 0.05, TBI + HBO1 groups vs. TBI + HBO2 groups

Rats in the TBI groups had higher mRNA levels of NF-κB p65 than the SH group rats, and rats post-treated with 1.6ATA HBO had a lower expression especially at 3 h and 6 h after TBI (*P* < 0.05) (Fig. 6). The protein levels of NF-κB p65 (Fig. 7) in the TBI groups were also significantly higher than in the SH groups, reaching a peak at 48 h, and declining by 1.6ATA HBO intervention at 12 h and 48 h respectively (*P* < 0.05). Nevertheless, there was no significant reduction through 2.2ATA HBO intervention.

We also compared different pressure treatments. The mRNA and protein levels of NF-κB p65 in the TBI + HBO1 groups were significantly lower than those of the TBI + HBO2 groups at 48 h (*P* < 0.05). Thus, it was concluded that 1.6ATA HBO attenuated the expression level of NF-κB p65 after TBI.

### 1.6ATA HBO reduced NF-κB-positive staining rates in the peri-lesioned brain tissue

**Fig. 8:**
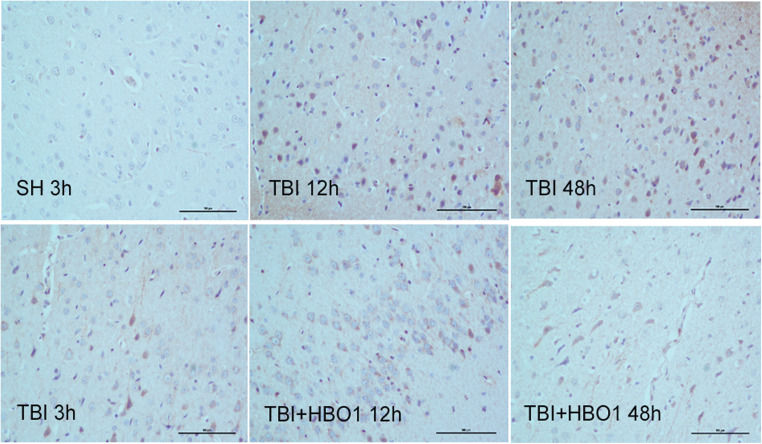
Representative images of immunohistochemistry staining of NF-κB in the peri-lesioned brain tissue (× 200). The brown stain was positive cell. Bar: 100 μm

Immunohistochemistry showed that high NF-κB expressions of injured brain tissue were observed from 3 h after TBI while there was almost no positive expression in the SH group. However, the NF-κB expression in the 12 and 48 h TBI + HBO1 groups was significantly reduced compared with the TBI group (*P* < 0.05) (Fig. 8 and Table 2). The NF-κB-positive staining rates of 1.6ATA HBO groups were significantly lower than the 2.2ATA HBO groups at 12 and 48 h. Combined with the results of Western blot, down-regulation of NF-κB indicated that 1.6ATA HBO protected rats against histone-induced immune damage via TLR4/MyD88/NF-κB signaling pathways following TBI.

**Table 2:** NF-κB-positive staining rates following TBI in experimental groups (% of total cells)

Group	SH	TBI	TBI + HBO1	TBI + HBO2
3 h	**9**.6 ± 2.7	12.7 ± 3.2	15.4 ± 2.9	17.3 ± 3.7
6 h	10.3 ± 2.1	13.9 ± 3.8	16.2 ± 3.1	20.5 ± 3.7
12 h	12.6 ± 4.2	32.8 ± 5.4^*^	16.7 ± 3.3^◆#^	29.5 ± 4.9^*^
24 h	14.3 ± 4.9	26.7 ± 6.2	24.1 ± 5.5	32.7 ± 7.2^*^
48 h	10.6 ± 3.5	55.6 ± 7.7^*^	29.5 ± 7.1^*◆#^	46.2 ± 5.9^*^

Data are presented as the mean ± standard deviation. *P < 0.05, TBI, TBI + HBO1, and TBI + HBO1 groups vs. SH groups; ^♦^*P* < 0.05, TBI + HBO1 and TBI + HBO2 groups vs. TBI groups; ^#^*P* < 0.05, TBI + HBO1 groups vs. TBI + HBO2 groups

### 1.6ATA HBO restrained extracellular histones-induced apoptosis in the peri-lesioned brain tissue

**Fig. 9:**
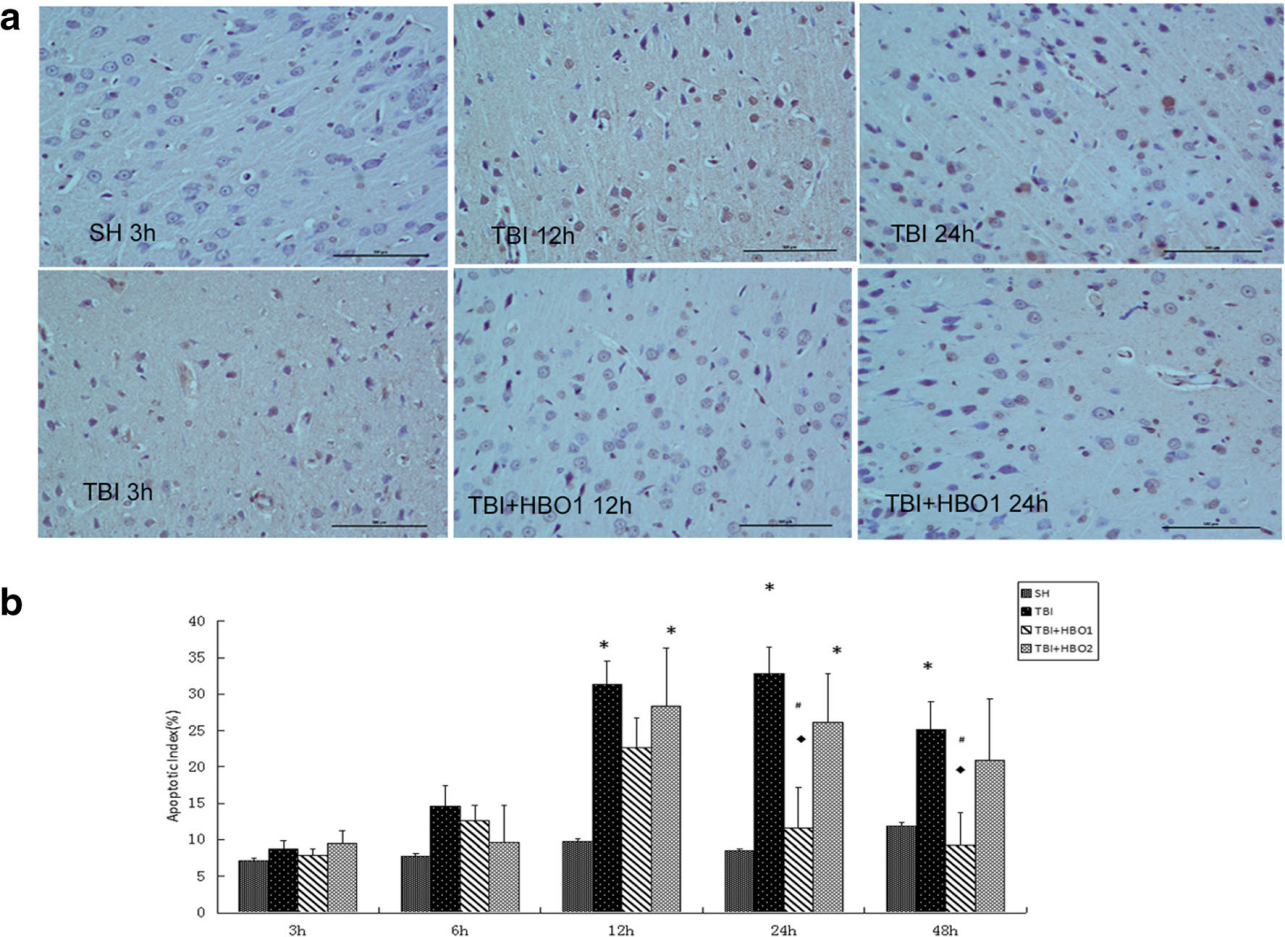
The effects of different ATA HBOT on the histone-induced apoptosis after TBI. (**a**) Representative images of TUNEL staining in the peri-lesioned brain tissue (× 200). The brown stain was a positive cell. Bar: 100 μm. (**b**) Comparison of apoptotic indexs of experimental groups.*P < 0.05, TBI, TBI + HBO1, and TBI + HBO1 groups vs. SH groups; ♦ *P* < 0.05, TBI + HBO1, and TBI + HBO2 groups vs. TBI groups; ^#^*P* < 0.05, TBI + HBO1 groups vs. TBI + HBO2 groups

Apoptotic cell death of neurons are hallmarks of secondary brain injury. In order to assess the effects of different ATA HBOT on extracellular histones-induced apoptosis, we analyzed the apoptotic index of each group. As shown in Fig. 9, the percentage of TUNEL-positive neurons was significantly higher in the TBI groups than in the SH groups (*P* < 0.05), whereas 1.6ATA HBO administration significantly decreased the percentage of TUNEL-positive neurons, especially at 24 h and 48 h following TBI (*P* < 0.05).

## Discussion

Previous studies have mostly described the influence of extracellular histones on cerebral vascular events and neurodegeneration of the central nervous system (CNS). The experiments in vivo animals conducted by De Meyer SF(De Meyer et al., 2012) showed models of ischaemic stroke presenting a sevenfold serum nucleosome increase. Jamison J et al. (Grailer & Ward, 2014) also observed that extracellular histone levels were elevated from C5a-induced models of ALI in mice, resulting in fibrin deposits, and epithelial cell injury. Nevertheless, they could not confirm the source of the extracellular histones release. In the current study, we dynamically detected the presence of extracellular histones in injured tissue samples from rats with TBI. The mRNA expression of extracellular histones in cortex tissue appeared to increase early (3–6 h). The proteins level sustained high expression to a peak value at 48 h. We speculated firstly that histones were passively released from dying nerve cells following TBI, which causes direct cytotoxicity to epithelial and endothelial of damaged peripheral tissue. Direct endothelial toxicity increases the permeability of cytomembrane, leukocyte migration, and aggregation.(Holdenrieder et al., 2001; Remijsen et al., 2011; Chu et al., 2014) Neutrophils might release extracellular web-like structures containing histones called neutrophil extracellular traps (NETs). In the process of NET formation (NETosis), histones could actively release from cells via decondensation of nuclear chromatin. In a previous study pre-incubation of NET with antibodies against histone H2 and H4 reduced NET-mediated cytotoxicity, suggesting histones are responsible for NET-mediated cytotoxicity.(Saffarzadeh et al., 2012) These results were in line with our observation on individual changes of histone HI, H2A, and H4 after TBI.

HBOT has been shown to promote neuronal survival, decrease the apoptosis of brain injury via a reduction in oxidative stress to modulate neuroinflammation.(Liu et al., 2014). Our results showed that HBO intervention could downregulate the expression of histones H1, H2A, H4 induced by TBI, while the decline in 1.6ATA groups was significantly lower than that in the 2.2ATA group, especially within 6 h. It confirmed preliminarily that 1.6ATA HBO might reduce secondary damage of TBI through inhibiting histone release more efficiently than 2.2ATA HBO.

It is known that histones also act as a DAMP protein alerting the body to neuron apoptosis, by activating immune processes through TLR4/MyD88/NF-κB signaling pathways. In our experiment, the effect of different pressure HBO on the expression of downstream NF-κB was another research hotspot. We examined the expression of NF-κB after TBI. Our results showed that the mRNA and protein levels of NF-κB were significantly increased in the early stage of TBI and that 1.6ATA HBO could downregulate their expression significantly in the 3 h, 12 h, and 48 h groups. In addition, we found that HBO also reduced the level of NF-κB p65, especially in the HBO 1.6ATA group. In addition, from the histologic analysis, the dynamic tendency of NF-κB immunohistochemistry stain was consistent with the pathological changes of apoptosis in the peri-lesioned brain tissue of experimental rats. It was inferred from data that 1.6ATA HBO could block histones interact with TLR4, inhibiting MyD88 signal transduction pathway, thereby attenuating expression level of NF-κB p65, which could downregulate proinflammatory cytokines transcription and nerve cell apoptosis in the secondary brain injury process (Buchanan et al., 2010).

It is interesting to note that in the TBI + HBO1 groups, not only the expression level of histones was significantly lowerbut also the mNSS scores of rats were higher than those in TBI + HBO2 groups, confirming that 1.6ATA HBO intervention was superior to 2.2ATA for neurologic impairment restoration after TBI. Why are lower atmosphere absolute hyperbaric oxygen treatments more effective? Previous studies showed that prolonged HBOT increased reactive oxygen species (ROS) formation, inducing adverse clinical outcomes (Bitterman, 2012). Hussein J. Hamam et al. considered acetylation of histone was a key component of NETosis which subsequently induced chromatin decondensation and deacetylase inhibitors (HDACis) required ROS production (Khan & Palaniyar, 2017; Hamam et al., 2019). Thus, the appropriate administration of HBO was crucial. It is our opinion that appropriate HBOT means proper therapeutic pressure (evaluation of brain tissue oxygen tension maintaining between 25 and 30 mmHg) (Narotam, 2013) and duration of HBOT (not more than once a day). Appropriate oxygen supplementation could inhibit the imbalance between ROS and antioxidants after TBI. It was demonstrated that proper administration of HBO might preserve the mitochondrial integrity confronted with oxidative stress caused by ischemia and hypoxia following TBI, decreasing the release of a burst of ROS from within the mitochondria (Hiebert et al., 2015). Combined with our data, we inferred that a relatively lower pressure HBO decreased production of ROS more efficiently, restrained histone modification and extensive chromatin decondensation, and eventually inhibited histones release to extracellular space.

## Conclusion

This study suggested for the first time that 1.6ATA HBO has an important protective role in inhibiting both the cytotoxic and the pro-inflammatory actions of histones in the early stage of secondary brain injury. The current study provides new ideas on the choice of the optimal onset time and pressure of HBO exposure in TBI therapy.
